# Identification of the Resveratrol Potential Targets in the Treatment of Osteoarthritis

**DOI:** 10.1155/2021/9911286

**Published:** 2021-12-07

**Authors:** Meng Zhou, Dacheng Wang, Jing Tang

**Affiliations:** ^1^Department of Sports Medicine, Beijing Jishuitan Hospital, Beijing 100035, China; ^2^Department of Orthopedics Surgery, Beijing Jishuitan Hospital, Beijing 100035, China

## Abstract

**Objectives:**

Osteoarthritis (OA) is a chronic joint degenerative disease and has become an important health problem for the elderly. However, there is still a lack of effective drugs for the treatment of OA. Our research combines bioinformatics and experimental strategies to determine the target of resveratrol for OA treatment.

**Methods:**

First, the differentially expressed genes (DEGs) of OA joint tissues were obtained from the related microarray gene expression data. Second, resveratrol, a natural polyphenol compound, was used to screen the drug treatment target genes. Third, the drug-disease network was established, and the resveratrol target genes for OA treatment were obtained and verified through experimental verification.

**Results:**

A total of 300 differentially expressed genes with 246 upregulated and 54 downregulated were found in OA joint tissues, and 310 resveratrol potential target genes were obtained. Finally, six genes, namely, CXCL1, HIF1A, IL-6, MMP3, NOX4, and PTGS2, were selected to validate the treatment effects of the resveratrol. The results showed that all six genes in human OA chondrocytes were significantly increased. In addition, in these chondrocytes, CXCL1, HIF1A, IL-6, MMP3, NOX4, and PTGS2 were reduced considerably, but HIF1A was significantly increased after resveratrol treatment.

**Conclusions:**

Our data indicates that CXCL1, HIF1A, IL-6, MMP3, NOX4, and PTGS2 are all targets of resveratrol therapy. Our findings may provide valuable information for the mechanism and therapeutic of OA.

## 1. Introduction

Osteoarthritis (OA) is a common chronic joint degenerative disease that causes limited joint mobility and chronic pain in the elderly [1], which affects the quality of life in many ways [2]. Many factors, including obesity, joint damage, deformity, trauma, and age, are responsible for the OA pathogenesis [3], which are mainly attributed to the gradual deterioration of cartilage structure and function [4]. Current OA treatment strategies, such as exercise, loss of weight, using crutches, and taking nonsteroidal anti-inflammatory drugs (NSAIDs) and analgesics, involve reducing pain and inflammation, limiting the loss of functional capacity, and maintaining joint mobility [5, 6]. However, for severe pain and adverse reactions to nonsurgical treatments, joint replacement is still the ultimate treatment option [7], and there is still an urgent need for more effective OA treatment methods.

OA is characterized by decreased extracellular matrix (ECM) synthesis and loss of tissue cellularity [8]. Chondrocytes are the only cellular components of cartilage [9], which can produce type II collagen and proteoglycan that constitute ECM, and play an important role in cartilage function [10]. Therefore, maintaining the normal function of chondrocytes is an essential factor in preventing the degeneration of cartilage.

Resveratrol (3,4,5-trihydroxystilbene) is a natural polyphenolic compound that can commonly be detected in most grape cultivars. Resveratrol has multiple functions, including antioxidant, antiapoptotic, and anti-inflammatory effects [11]. Resveratrol has a protective therapeutic effect on aging diseases, such as heart disease and diabetes. In addition, resveratrol can ameliorate OA symptoms by inhibiting the activation of nuclear factor-kB (NF-kB) [12] and then downregulating NF-kB-induced catabolism-related genes [13]. Previous studies showed that intra-articular injection of resveratrol could prevent the destruction of OA cartilage [14], delayed articular cartilage degeneration, and promoted chondrocyte autophagy by regulating AMPK/mTOR signaling pathway [15] in surgery-induced OA mouse models. Although many studies have been conducted on the biological activity of resveratrol, its mechanism of action on OA is still not fully understood.

In this study, we used the comprehensive systems pharmacology methods to explore the possible mechanisms of resveratrol in OA treatment. We first obtained the OA-related DEGs and resveratrol target genes, and then the OA-related resveratrol target genes were obtained and verified by quantitative polymerase chain reaction (qPCR) and western blotting in resveratrol-treated human OA chondrocytes. Our research provides a more efficient way to illustrate the therapeutic mechanism of resveratrol on osteoarthritis.

## 2. Materials and Methods

### 2.1. Osteoarthritis-Related Genes Analysis

The expression data of GSE41342 was obtained from the study of “data from a time course study of gene expression in a mouse model of osteoarthritis” in the Gene Expression Omnibus (GEO) database (https://www.ncbi.nlm.nih.gov/geo/). This study aims to find DEGs of the OA joint tissues from normal control (NC) mice [16], which is consistent with our study. DEGs were analyzed and obtained by the NetworkAnalyst platform [17], *p* < 0.05 and ｜logFC｜ >1 as cut-off values. The DEGs were represented by a volcano plot, and gene expression data was represented by a heatmap generated by ClustVis online tools (https://biit.cs.ut.ee/clustvis/). The expression data of the DEGs were obtained from the NetworkAnalyst platform.

### 2.2. Resveratrol Target Genes Identification and Network Establishment

The chemical information of resveratrol was obtained from the PubChem database (https://pubchem.ncbi.nlm.nih.gov/). Afterward, the resveratrol target genes were screened from the Comparative Toxicogenomics Database (CTD) [18] and the similarity ensemble approach (SEA) database (http://sea.bkslab.org/), and the target gene functions were analyzed by the Swiss Target Prediction database (http://www.swisstargetprediction.ch/). The Venn diagram obtained the overlap of resveratrol target genes and OA DEGs. Cytoscape 3.8.2 was used to draw the gene networks.

### 2.3. The Gene Ontology Analysis

The Gene Ontology (GO) and Kyoto Encyclopedia of Genes and Genomes (KEGG) enrichment analysis were using the Metascape database, which provides a biologist-oriented resource for the analysis of systems-level datasets [19]. The target genes were mapped into the Metascape database and to identify the biological processes and KEGG pathways of the involved genes. Furthermore, the protein-protein interactions (PPIs) of the genes were analyzed by the STRING database (https://string-db.org/), and the link of the genes was calculated by CytoHubba application in Cytoscape software.

### 2.4. Chondrocyte Isolation and Treatment

Adult human osteoarthritic articular cartilage samples were obtained from patients admitted to the Department of Orthopedics of Jishuitan Hospital (Beijing, China) with OA undergoing total knee replacement (*n* = 3). The articular cartilage samples were treated with collagenase type II and type IV (1 mg/ml each) in 15 ml Dulbecco's Modified Eagle Medium (DMEM) supplemented with 10% fetal bovine serum (FBS) and 1% penicillin and streptomycin (Life Technologies, Carlsbad, California) and then incubated at 37°C in 5% carbon dioxide overnight. Single cells were filtered through a nylon mesh filter and collected by centrifugation and then washed three times with 10 ml phosphate-buffered saline (PBS). The collected OA chondrocytes were then plated at a density of 1 × 10^5^ cells/cm^2^. Normal human chondrocytes were obtained from the National Infrastructure of Cell Line Resource (Beijing, China). Each group of chondrocytes was cultivated for 48 h separately prior to subsequent experiments. Ten µM resveratrol (Sigma, Burlington, MA) was added to the cells, followed by incubation for 24 h. The treatment cells were then harvested and prepared for the following tests. All procedures performed in the study have been approved by the Ethics Committee of Jishuitan Hospital, and the informed consent of all individuals participating in the study or their guardians has been obtained.

### 2.5. Quantitative PCR (qPCR) Analysis

The drug-treated cells were collected and washed by PBS, and then using the RNeasy Mini Kit (Qiagen, Germany) the total RNA is extracted and reversed to cDNA by Bio-Rad RT-PCR System (Hercules, CA, USA). Follow the instructions of the Talent qPCR kit (TIANGEN, Beijing, China) to configure the reaction mixtures, and then incubate it at 95°C for 5 min, followed by 40 cycles of 95°C for 15 s and 60°C for 1 min using the ABI 7500 Fast System (Applied Biosystems, New York, USA). Data were normalized to GAPDH. The primers used in this study are shown in Table 1.

### 2.6. Western Blot Analysis

The cells were homogenized in radio-immunoprecipitation assay (RIPA) lysis buffer containing protease and phosphatase inhibitors (Solarbio, Beijing, China) and centrifuged at 12,000 rpm for 30 min at 4°C. The supernatant was mixed with 4x loading buffer and heated to 100°C for 5 minutes to denature the proteins. Protein concentrations were determined using the bicinchoninic acid (BCA) protein assay (Solarbio, Beijing, China). Protein samples (30 *µ*g each) were electrophoretically separated by sodium dodecyl sulphate polyacrylamide gel electrophoresis (SDS-PAGE) before transferring to polyvinylidene fluoride (PVDF) membranes (0.20 *μ*m; Millipore, Billerica, MA, USA). The membranes were incubated with bovine serum antibody (BSA) for 1 h at room temperature. Afterwards, the blots were incubated with different primary antibodies, anti-CXCL1 (1 : 1000; Abcam, Cambridge, UK), anti-HIF1A (1 : 1000; Abcam), anti-IL-6 (1 : 1000; Abcam), anti-MMP3 (1 : 1000; Abcam), anti-NOX4 (1 : 1000; Abcam), anti-PTGS2 (1 : 1000; Abcam), anti-Collagen II (1 : 1000; Abcam), anti-aggrecan (1 : 1000; Abcam), and anti-GAPDH (1 : 1000; Abcam), overnight at 4°C. After being washed with Tris-buffered saline and Tween 20 (TBST), the blots were incubated with the secondary antibodies, IgG-HRP (1 : 10000; Abcam), for one h at room temperature. Band signal was visualized by the enhanced chemiluminescent (ECL) kit (Invitrogen, Carlsbad, CA, USA) and detected by Bio-Rad fluorescence scanner. The resulting images of the bands in question were visualized by Image-Lab software.

### 2.7. Statistical Analysis

The relative protein expressions were quantified by Image J software (NIH, shareware). The data from the qPCR or western blot tests were calculated by GraphPad Prism software version 8.0 (CA, USA). The results are expressed as the Mean ± SEM. All data were analyzed by two-tailed Student's *t*-test. A *p*-value of less than 0.05 was considered statistically significant.

## 3. Results

### 3.1. Obtain Osteoarthritis-Related Differentially Expressed Genes

**Figure 1:**
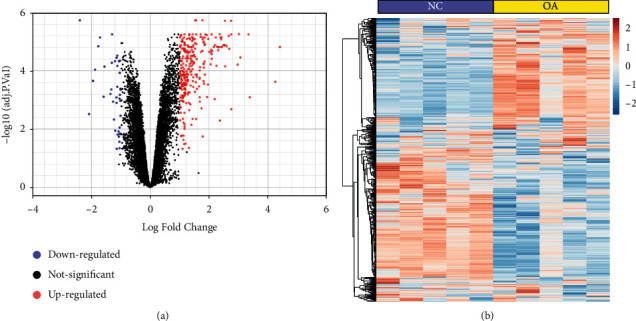
The differentially expressed genes (DEGs) of the osteoarthritis (OA) joint tissues. (a) The volcano plot of DEGs in the joint tissues between the normal control (NC) group and the OA group. (b) Heatmaps of DEGs in the joint tissues of the NC group and the OA group. Colors in the heatmaps indicate the Row Z-score among the different datasets. The red and blue nodes represent high expression and low expression genes, respectively.

The OA mouse model joint tissue gene expression dataset GSE41342 was obtained from the GEO database to analyze the genes related to OA. The gene expression comparison was made on the normal control (NC) group and osteoarthritis group. As shown in Figure 1, there were a total of 300 differentially expressed genes with 246 upregulated and 54 downregulated in OA joint tissues, which may be closely related to the progression of OA.

### 3.2. Prediction of OA-Related Resveratrol Target Genes by Comprehensive Systems Pharmacology Methods

**Figure 2:**
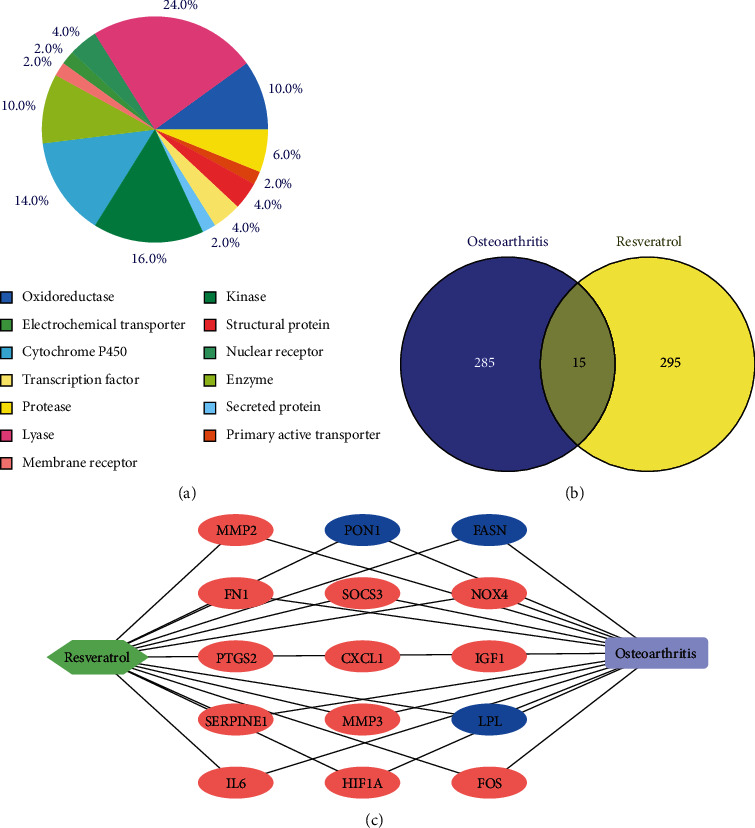
The resveratrol potential target genes in OA treatment. (a) The classification is based on the biochemical criteria of drug target genes. Each item is shown in different colors. (b) Venn diagram of resveratrol target genes and OA DEGs. (c) The resveratrol-target genes-OA network. The red and blue nodes represent upregulated and downregulated genes, respectively.

Based on the resveratrol chemical information, the CTD and SEA databases were used to predict the resveratrol potential target genes. Finally, a total of 310 genes were obtained from those databases. Then, according to the biochemical criteria, the function of the target genes was classified, as shown in Figure 2(a). These 310 targets mainly include lyase, kinase, cytochrome P450, oxidoreductase, and enzyme.

To further analyze the resveratrol's effects on OA, the overlap of drug target genes and OA DEGs was shown as a Venn diagram, which was 15 genes in both groups (Figure 2(b)). The overlap genes were not only OA-related genes but also the resveratrol treatment targets. In addition, a network of resveratrol target gene osteoarthritis was constructed (Figure 2(c)). Among them, 12 genes are upregulated, and three genes are downregulated, which indicated that resveratrol might have an effect on OA by stimulating or inhibiting these target genes.

### 3.3. The Target Gene Functional Analysis

**Figure 3:**
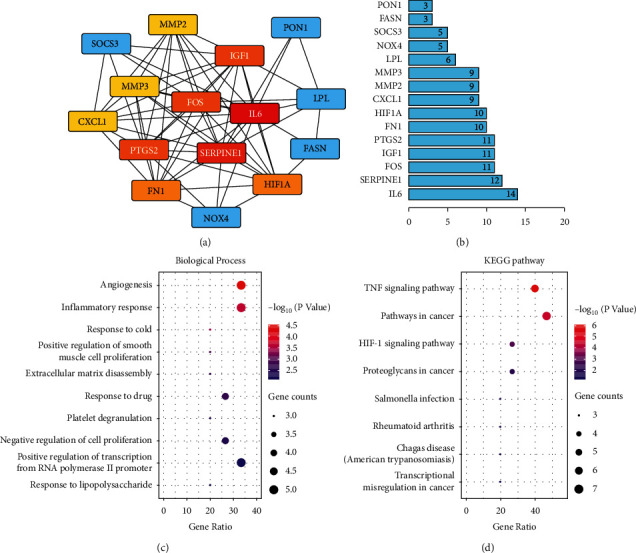
The gene function of the potential target genes. (a) The target genes protein-protein interaction (PPI) network. The hub genes are represented as red and yellow nodes. A deeper red color indicates more connections. (b) Bar plot of the number of hub gene links. (c) The biological processes of the target genes Gene Ontology (GO) analysis. (d) The KEGG pathway of the target genes. Circle size represented the number of genes involved in each GO or KEGG term, and different colors defined by the *p*-value.

In order to further clarify the connection between these target genes, the PPI analysis was conducted by the STRING database. The PPI network was generated, and the link of the genes was calculated. The hub genes in the network were marked in red and yellow, and other genes related to the hub genes were represented by blue nodes (Figure 3(a)). A bar plot of the number of hub gene links is shown in Figure 3(b), and the genes, such as IL-6, PTGS2, HIF1A, CXCL1, and MMP3, may play a critical role in the biological activity in OA progression and resveratrol treatment.

Furthermore, to gain a comprehensive understanding of these target genes, the GO and KEGG gene function analysis was conducted by the Matescape database. Fifty-five biological processes GO items and 12 KEGG pathways were enriched. The biological processes such as inflammatory response and extracellular matrix disassembly (Figure 3(c)) and KEGG pathways such as TNF signaling pathway and HIF-1 signaling pathway (Figure 3(d)) may be involved in the process of drug treatment of disease.

### 3.4. The Expression Changes of Selected Resveratrol Target Genes in OA

**Figure 4:**
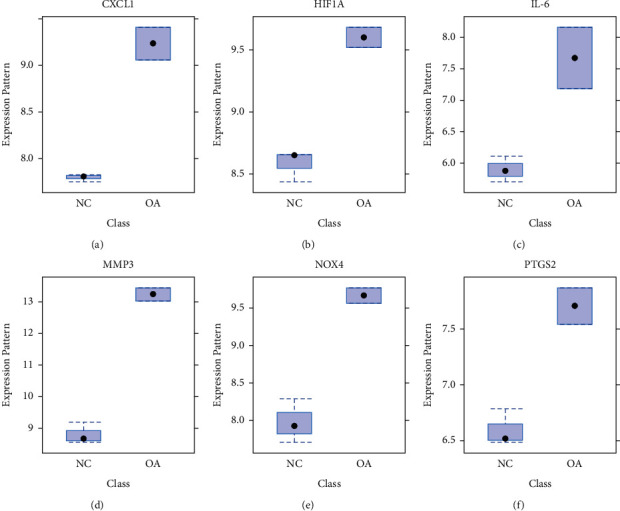
The gene expression pattern in NC and OA group of GSE41342 dataset. The expression of CXCL1 (a), HIF1A (b), IL-6 (c), MMP3 (d), NOX4 (e), and PTGS2 (f) in different groups. Values represented as Mean ± SEM (*n* = 5 in each group).

**Figure 5:**
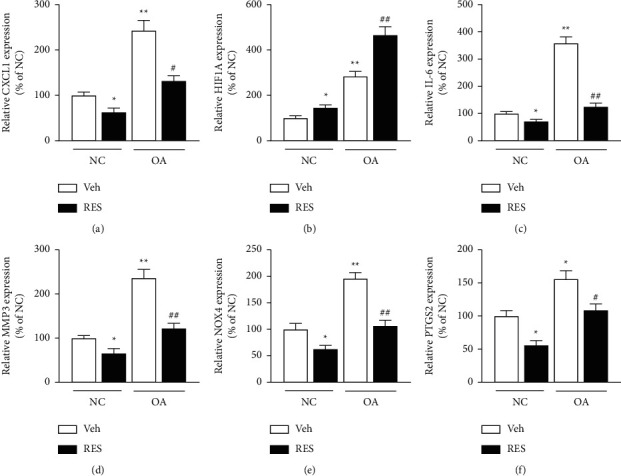
The gene expression among different groups of human chondrocytes by qPCR analysis. The mRNA expression level of CXCL1 (a), HIF1A (b), IL-6 (c), MMP3 (d), NOX4 (e), and PTGS2 (f) in different groups. Data were normalized to GAPDH, and values represented as Mean ± SEM (*n* = 5 in each group). ^*∗*^*p* < 0.05,  ^*∗∗*^*p* < 0.01 vs. NC + Vehicle group, ^#^*p* < 0.05, ^##^*p* < 0.01 vs. OA + Vehicle. Veh, Vehicle; RES, resveratrol.

**Figure 6:**
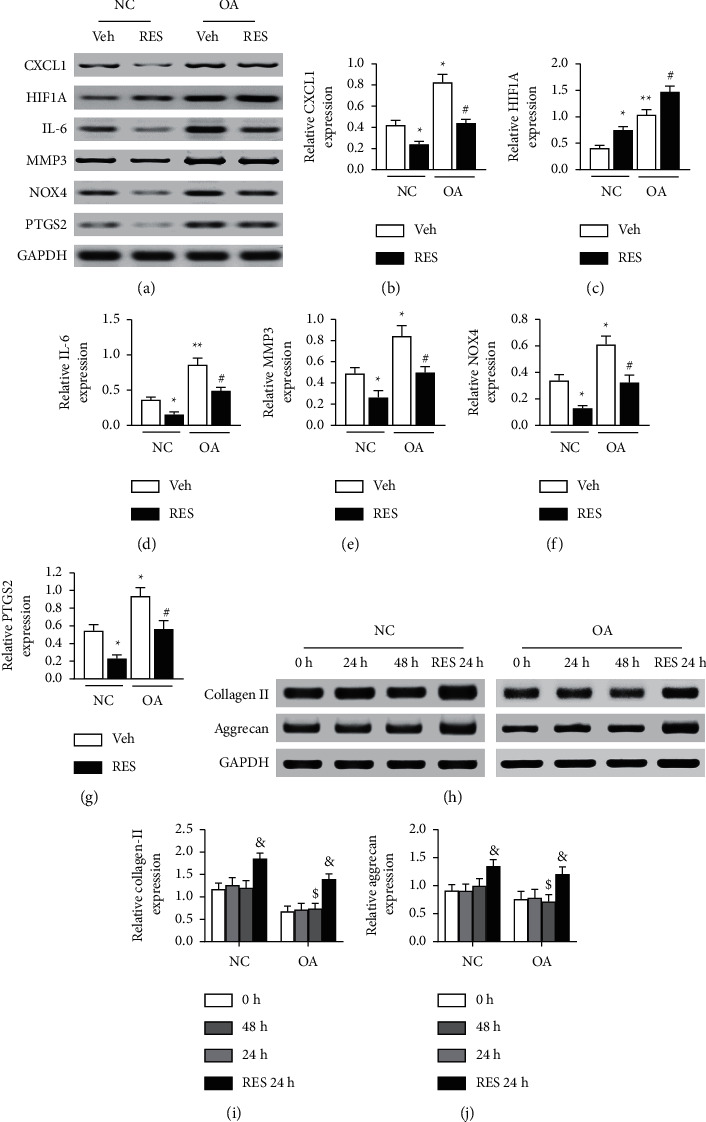
The gene expression among different groups of human chondrocytes by western blot analysis. (a–g) The western blot bands (a) and relative protein expression levels of CXCL1 (b), HIF1A (c), IL-6 (d), MMP3 (e), NOX4 (f), and PTGS2 (g) in different groups. (h–j) The western blot bands (h) and relative protein expression levels of collagen II (i) and aggrecan (j) at different time points. Data were normalized to GAPDH, and values represented as Mean ± SEM (*n* = 3 in each group). ^*∗*^*p* < 0.05,  ^*∗∗*^*p* < 0.01 vs. NC + Vehicle group, ^#^*p* < 0.05 vs. OA + Vehicle, ^&^*p* < 0.055 vs. 0 h ^$^*p* < 0.05 vs. NC-48 h Veh, Vehicle; RES, resveratrol.

Based on the comprehensive systems pharmacology and gene function analysis, six genes, including CXCL1, HIF1A, IL-6, MMP3, NOX4, and PTGS2, were selected to validate the treatment effects of the resveratrol. In the GSE41342 dataset, all six genes are significantly upregulated in the OA group (Figure 4). To further validate our findings, a series of qPCR quantification and western blot testing were performed in the human OA chondrocytes. As shown in Figures 5 and 6, the mRNA and protein levels of all six genes were significantly increased (*p* < 0.05) in human OA chondrocytes, consistent with the data analysis results. Moreover, the mRNA and protein levels of CXCL1, IL-6, MMP3, NOX4, and PTGS2 were significantly decreased (Figures 5(a), 5(c)–5(f), 6(a), and 6(c)–6(f), *p* < 0.05), but HIF1A was increased considerably (Figures 5(b) and 6(b), *p* < 0.05), after treatment of cells with resveratrol for 24 h. The above results indicate that all six genes are closely related to the OA process and can be used as therapeutic targets for resveratrol treatment. In addition, there are no significant changes in human chondrocyte phenotype protein collagen II and aggrecan (Figures 6(h)–6(j)) before the drug treatment, but they significantly decreased in OA group. After being treated with resveratrol, the collagen II and aggrecan are significantly increased in both NC and OA group, which indicated that the human chondrocytes cultured in our study maintained the chondrocyte phenotype throughout the experiment and can be promoted by resveratrol treatment.

## 4. Discussion

Resveratrol is a natural phytoalexin, and many studies have demonstrated its potential therapeutic effect on OA by regulating chondrocyte metabolism, apoptosis, and proliferation [20, 21]. To further clarify the possible mechanism, we used the bioinformatics analysis combined with subsequent experimental verification to investigate the mechanism of resveratrol in the treatment of osteoarthritis. Based on the bioinformatics analysis strategy, we obtained 15 DEGs closely related to OA pathology and resveratrol treatment (Figure 2). The enrichment analysis indicated that the DEGs were highly enriched in multiple GO terms and KEGG signaling pathways, including inflammatory response, extracellular matrix disassembly, TNF signaling pathway, and HIF-1 signaling pathway, which may be involved in the biological activity in the resveratrol treatment process (Figure 3). Previous study has shown that resveratrol can improve cartilage damage by inhibiting the levels and expressions of inflammatory mediators, and then regulating the inflammatory-dependent signaling pathways [22]. Moreover, the attrition of the extracellular matrix (ECM) can dramatically alter the structure and function of joints and result in the development of OA [23], which is consistent with our findings. The GO terms and KEGG signaling pathways may provide valuable information for OA pathology and resveratrol treatment.

It is increasingly understood that the role of resveratrol depends on different OA models and administration methods. *In vivo* studies have shown that oral resveratrol can inhibit the development of OA by decreasing body weight, reducing degradation of type II collagen, and suppressing chondrocyte apoptosis [24]. In addition, resveratrol intra-articular injection delayed cartilage degeneration through promoting chondrocyte autophagy in the destabilization of the medial meniscus- (DMM-) induced OA model, in part via regulating HIF-1*α*-dependent AMPK and mTOR signaling [15]. *In vitro* studies have shown that resveratrol treatment of OA chondrocytes can inhibit various inflammatory signal pathways [25], protect cells from apoptosis [13], and reduce the production of advanced glycation end products and matrix metalloproteinases [26]. In our study, we collected chondrocytes from articular cartilage of patients with OA, and then the changes of cell phenotype proteins and DEGs were observed after treatment with resveratrol. We found that the human chondrocyte phenotype protein collagen II and aggrecan significantly decreased in OA group but can be promoted by resveratrol treatment, consistent with previous studies.

After analyzing the DEGs by functional enrichment analysis and literature review, six genes, including CXCL1, HIF1A, IL-6, MMP3, NOX4, and PTGS2, were chosen for experimental validation. We found that all six genes were significantly changed in human OA chondrocytes. Meanwhile, all six genes were markedly changed after resveratrol treatment, suggesting that they may serve as the crucial targets in the progression of OA and resveratrol treatment.

Chemokine (CXC motif) ligand 1 (CXCL1), also known as growth-regulated protein a (GROa), belongs to the ELR-family of chemokines and modulates angiogenesis, inflammation, and tumorigenesis [27, 28]. Previous studies have reported that CXCL1 contributes to the ingress of neutrophils into rheumatoid arthritis (RA) joint [29], and during cartilage development, CXCL1 induces chondrocyte hypertrophy and apoptosis [30]. In general, CXCL1 levels are low under normal physiological conditions but appear to be elevated in patients with RA and OA [31]. In this study, we predicted and verified that the level of CXCL1 was significantly increased in the joint tissues of OA model mice and human OA chondrocytes, which is consistent with the previous studies. Moreover, resveratrol treatment can inhibit the expression of CXCL1, indicating that CXCL1 is the target of resveratrol treatment in patients with OA. According to this evidence, CXCL1 may play a pivotal role in the pathogenesis and treatment of OA.

Hypoxia-inducible factor 1 (HIF-1) belongs to the family of basic-helix–loop–helix-containing PAS domain transcription factors and consists of the subunits HIF-1*α* and HIF-1*β*, produced in response to hypoxia [32]. HIF-1*α* is well known as one of the major regulators of the hypoxic response [33] and controls hypoxic expression of erythropoietin, as well as the expression of genes with metabolic functions [34], which is essential in tumor genesis and inflammation [35]. Our previous study showed that HIF-1*α* is upregulated in bone tissue of rats treated with hind limb occlusion, which plays an essential role in fracture healing [36]. It has been demonstrated that HIF-1*α* is expressed in OA articular cartilage [37] and plays a significant role in chondrocyte survival [38]. HIF-1*α* could alleviate apoptosis and senescence via mitophagy in chondrocytes under hypoxia conditions, which could also ameliorate surgery-induced cartilage degradation in mice OA model [39]. However, the association between HIF-1*α* and OA has not been sufficiently explored, and HIF-1*α* regulation in OA progression is poorly understood. In the present study, our results demonstrated that HIF-1*α* expression is increased in the joint tissues of OA model mice and human OA chondrocytes and can be enhanced by resveratrol treatment, which may be involved in the HIF-1 signaling pathway. These findings suggest that the resveratrol may stimulate the HIF-1*α* to promote the matrix accumulation and decrease degradation of human OA chondrocytes, indicating that HIF-1*α* may serve as a promising strategy for OA treatment.

Interleukin-6 (IL-6) is known as a mediator of inflammation, immune response, and hematopoiesis [40]. Due to the pathological effects of IL-6 under many adverse conditions, targeting IL-6 has become important in drug development. IL-6 is detected in synovial fluid and expressed in osteoarthritic cartilage and has an essential role in the pathogenesis of OA [41], which makes its inhibition a potential target in the OA treatment [42]. A previous review has confirmed the critical role of IL-6 in the progression of OA, which is supported by the finding that IL-6 expression is elevated in OA human synovial fluid and sera [43]. CXCL1/CXCR2 activation promoted IL-6 expression in a time-dependent manner [44], suggesting IL-6 is an immediate-early gene in OA pathogenesis. In the present study, IL-6 was significantly increased in the joint tissues of OA model mice and human OA chondrocytes, which can be inhibited after resveratrol treatment, which may be involved in the TNF signaling pathway. This indicates that IL-6 has the function to promote the OA process as the target for resveratrol treatment. Although the function of IL-6 in OA has been proposed, the role of IL-6 in OA still requires further studies.

Matrix metalloproteinases (MMPs) are extracellular zinc-dependent proteases. It not only has a crucial role in extracellular decomposition processes [45] but also provokes focal destruction of the vascular ECM through proteolysis [46]. MMP3 is a member of the MMPs family and a joint modulator of ECM involved in disease morphogenesis, wound healing, tissue repair, and remodeling [47]. Ma et al. showed that the expression levels of serum MMP3 and inflammatory mediators in rheumatoid arthritis were positively correlated with the severity of the disease [48]. Also, MMP3 was found to be overexpressed in OA synovial cells, and high expression of MMP3 would promote cell proliferation and inhibit cell apoptosis, increasing OA inflammation [49]. Based on the important function in bone-related diseases, MMP3 was chosen as the candidate genes to investigate the OA progress and resveratrol treatment. In the present study, MMP3 was significantly increased in the joint tissues of OA model mice and human OA chondrocytes, which can be reversed by resveratrol treatment. The present study not only provided a treatment strategy for OA but also proved that MMP3 might be a new biomarker for judging the prognosis of OA.

NADPH Oxidase (NOX) 4, a member of the NOX family, is the source of reactive oxygen species (ROS) production [50]. Temporal and spatially directed specific inhibition of abnormal ROS signals in the inflammatory response after joint injury may have the potential to protect normal joint tissues and reduce early changes associated with the development of osteoarthritis [51]. NOX4 constitutively produces hydrogen peroxide (H_2_O_2_) [52] and participates in the development and homeostasis of tissues that make up normal joints [53]. NOX4 plays a significant role in the acute phase after joint injury, and targeted inhibition of inflammation caused by NOX4 may help prevent early joint changes in the pathogenesis of posttraumatic osteoarthritis [54]. However, the role of NOX4 in the OA progress and resveratrol treatment remains blank. In this study, we firstly demonstrated that NOX4 significantly increased human OA chondrocytes, and it was also the target of resveratrol treatment. An in-depth study of the role of NOX4 in the diagnosis and treatment of OA patients is essential and has aroused great interest.

Prostaglandin-endoperoxide synthase 2 (PTGS2) encodes cyclooxygenase-2, which is highly polymorphic and has a type of single nucleotide polymorphism located in its regulatory regions [55]. Previous studies have also identified high expression of PTGS2 in human OA chondrocytes and synovium samples [56] and showed increased expression in both mouse and human OA cartilage [57]. Downregulation of PTGS2 reduces the expression of IL-6 and MMP-3 [58], and the downregulation of MMP-3 and PTGS2 have been shown to help inhibit synovial fibroblast proliferation and joint inflammation in rheumatoid arthritis [59]. In the present study, PTGS2 was significantly increased in the joint tissues of OA model mice and human OA chondrocytes, which is consistent with the previous research. Moreover, resveratrol treatment can inhibit PTGS2 expression, which indicates that PTGS2 has the function to promote the OA process as a target for resveratrol treatment.

Our study provides valuable mechanism information of the resveratrol in the treatment of osteoarthritis, which is the first study to use such an approach for predicting resveratrol treatment OA targets. The limitation of our study is only using human OA chondrocytes to simulate the OA status. Alternative methods, such as using OA animal models, would further support our findings, and the predicted genes need to be validated on large-scale samples in the future.

## 5. Conclusion

Fifteen potential resveratrol target genes in the OA treatment were obtained from 310 resveratrol target genes and 300 OA-related genes. After functional analysis and experimental verification, six genes (CXCL1, HIF1A, IL-6, MMP3, NOX4, and PTGS2) are verified to be involved in the progression of OA and also can be used as the therapeutic target for OA treatment. Our study provides valuable information for the mechanism of OA pathogenesis and clinical treatment.

## Figures and Tables

**Table 1:** The primer sequences for real-time PCR.

Gene	Forward primer	Reverse primer
CXCL1	5′-CTGGGATTCACCTCAAGAACATC-3′	5′-CAGGGTCAAGGCAAGCCTC-3′
HIF1A	5′-GAACGTCGAAAAGAAAAGTCTCG-3′	5′-CCTTATCAAGATGCGAACTCACA-3′
IL-6	5′-ACTCACCTCTTCAGAACGAATTG-3′	5′-CCATCTTTGGAAGGTTCAGGTTG-3′
MMP3	5′-AGTCTTCCAATCCTACTGTTGCT-3′	5′-TCCCCGTCACCTCCAATCC-3′
NOX4	5′-CAGATGTTGGGGCTAGGATTG-3′	5′-GAGTGTTCGGCACATGGGTA-3′
PTGS2	5′-CTGGCGCTCAGCCATACAG-3′	5′-CGCACTTATACTGGTCAAATCCC-3′
GAPDH	5′-GGAGCGAGATCCCTCCAAAAT-3′	5′-GGCTGTTGTCATACTTCTCATGG-3′

## Data Availability

The analyzed data used to support the findings of this study are available from the corresponding author upon request.

## Abbreviations

OA: Osteoarthritis
DEGs: Differentially expressed genes
NSAIDs: Nonsteroidal anti-inflammatory drugs
ECM: Extracellular matrix
NF-kB: Nuclear factor-kB
qPCR: Quantitative polymerase chain reaction
NC: Normal control
GEO: Gene expression omnibus
CTD: Comparative Toxicogenomics database
SEA: Similarity ensemble approach
GO: Gene ontology
KEGG: Kyoto encyclopedia of genes and genomes
PPIs: Protein-protein interactions
DMEM: Dulbecco's Modified Eagle Medium
FBS: Fetal bovine serum
PBS: Phosphate-buffered saline
RIPA: Radio-immunoprecipitation assay
BCA: Bicinchoninic acid
SDS-PAGE: Sodium dodecyl sulphate polyacrylamide gel electrophoresis
PVDF: Polyvinylidene fluoride
BSA: Bovine serum antibody
TBST: Tris-buffered saline and tween 20
CXCL1: Chemokine (CXC motif) ligand 1
GROa: Growth-regulated protein a
HIF-1: Hypoxia-inducible factor 1
IL-6: Interleukin-6
MMPs: Matrix metalloproteinases
NOX: NADPH Oxidase
ROS: Reactive oxygen species
PTGS2: Prostaglandin-endoperoxide synthase 2.
